# Colonic bacterial diversity and dysbiosis in active microscopic colitis as compared to chronic diarrhoea and healthy controls: effect of polyethylene glycol after bowel lavage for colonoscopy

**DOI:** 10.1186/s12876-022-02392-w

**Published:** 2022-06-28

**Authors:** Lissette Batista, Virginia Robles, Chaysavanh Manichanh, Laura Ruiz, Danila Guagnozzi, Ferran Pinsach, Francisco Guarner, Fernando Fernández-Bañares

**Affiliations:** 1 Department of Gastroenterology, Hospital Universitari Mutua Terrassa, Plaza Dr Robert 5, 08221 Terrassa (Barcelona), Spain; 2 Department of Gastroenterology, Digestive System Research Unit, Institut de Recerca Vall d’Hebron, University Hospital Vall d’Hebron, Barcelona, Spain; 3 Centro de Investigación Biomédica en Red de enfermedades hepáticas y digestivas (CIBERehd), Instituto de Salud Carlos III, Madrid, Spain

**Keywords:** Microscopic colitis, Dysbiosis, Faecal microbiome, Polyethylene glycol

## Abstract

**Background:**

Most microbiota studies in microscopic colitis patients are performed after diagnostic colonoscopy without considering the potential effect of colonic lavage. Patients may achieve clinical remission after colonoscopy and it is unknown whether lavage-induced changes play a role.

**Aim:**

To assess the effect of polyethylene glycol (PEG) colonic lavage on clinical remission rate, microbial diversity, microbial dysbiosis index and specific microbial changes in patients with active microscopic colitis as compared to other diarrhoeal diseases and healthy controls.

**Methods:**

Fifty-five consecutive patients presenting chronic watery diarrhoea and 12 healthy controls were included. Faecal samples were collected three days before and 30 days after PEG in patients and controls for microbiome analysis.

**Results:**

Clinical remission was observed in 53% of microscopic colitis patients, and in 32% of non-microscopic colitis patients (*p* = 0.16). Considering patients with persisting diarrhoea after colonoscopy, 71% of non-microscopic colitis patients had bile acid diarrhoea. Baseline Shannon Index was lower in diarrhoea groups than in healthy controls (*p* = 0.0025); there were no differences between microscopic colitis, bile-acid diarrhoea and functional diarrhoea. The microbial dysbiosis index was significantly higher in microscopic colitis than in bile acid diarrhoea *plus* functional diarrhoea (*p* = 0.0095), but no bacterial species showed a significantly different relative abundance among the diarrheal groups.

**Conclusions:**

Dysbiosis is a feature in active microscopic colitis, but loss of microbial diversity was similar in all diarrheal groups, suggesting that faecal microbial changes are not due to microscopic colitis itself but associated with stool form. A considerable number of microscopic colitis patients achieved clinical remission after colonoscopy, but we were unable to demonstrate related PEG-induced changes in faecal microbiome.

**Supplementary Information:**

The online version contains supplementary material available at 10.1186/s12876-022-02392-w.

## Introduction

Microscopic colitis (MC) is an inflammatory bowel disease characterized by chronic non-bloody watery diarrhoea. The diagnosis is performed by microscopic examination of mucosal biopsies that reveal specific histopathological changes [1, 2]. There are two main types of MC: lymphocytic colitis (LC) and collagenous colitis (CC). MC most commonly presents in elderly and women. The pooled overall incidence rate of MC is estimated to be 11.4 (95% confidence interval 9.2–13.6) cases per 100,000 person-years [2].

The aetiology of MC is unknown and probably multifactorial and has been recently reviewed [3]. The current hypothesis supports the interrelation between luminal factors and both innate and adaptive mucosal immunity. This could induce gut barrier dysfunction and inflammation in the colonic mucosa [3]. It has long been hypothesized that the microbiome plays a key role in the pathogenesis of MC. In support of this, it has been shown that the faecal stream diversion in patients with CC refractory to standard therapy results in recovery from inflammation and histological remission, followed by disease relapse upon reconstruction of intestinal continuity [4]. Furthermore, two studies have demonstrated a reduced alpha diversity [5] and a higher microbial dysbiosis index [6] in patients with active MC compared with healthy controls. One study showed improved alpha diversity and faecal microbial composition reaching levels like healthy controls eight weeks after budesonide therapy [5]. However, this improvement occurred irrespective of clinical treatment response.

Studies characterizing specific microbial changes in MC have been small in sample size, and were, for the most part, cross-sectional and did not yield consistent findings [5–10]. These studies have recently been reviewed [3, 11]. In all of them, faecal samples for microbiota studies were obtained after diagnostic colonoscopy, without considering the effect of colonic lavage on the results. Bowel preparations affect the composition and diversity of the faecal and luminal microbiota in the short term, introducing potential bias into experiments examining the gut microbiota [12, 13]. Furthermore, there are a number of MC patients who achieve clinical remission after colonoscopy, but there are not prospective studies evaluating the remission rate after colonoscopy. Likewise, it is unknown whether colonic lavage induces gut microbiota changes that play a role in MC patients achieving clinical remission.

The aim of the present study was therefore to evaluate microbial diversity, the microbial dysbiosis index and specific microbial changes in faecal samples obtained before and after diagnostic colonoscopy in patients with active MC. In this sense, the effect of polyethylene glycol (PEG) colonic lavage on both the clinical remission rate and the gut microbiota was assessed. Two diarrheal control groups (functional diarrhoea -FD- and bile acid diarrhoea -BAD-) and one healthy control group were included for comparison.

## Patients and methods

### Study design

#### Study population

Consecutive patients with chronic non-bloody watery diarrhoea were prospectively included during the period from September 2014 to December 2018 based on the following inclusion and exclusion criteria, which were selected to maximize the pre-test probability of MC.

- Inclusion criteria: (1) Women 50 years or older and men 70 years or older; (2) chronic watery diarrhoea with two or more daily liquid stools (Bristol scale = 6 or 7) or frequent episodes (at least three times a week) of watery diarrhoea (Bristol scale = 6 or 7), with a duration of at least one month; (3) normal blood test and biochemistry (including C reactive protein and TSH), negative anti-transglutaminase antibodies, and negative faecal ova and parasites; (4) patients with an indication for a diagnostic colonoscopy by their physician at charge, mainly to rule out MC; and (5) signature of the study informed consent.
- Exclusion criteria: (1) patients with alternating diarrhoea-constipation and self-limiting diarrhoea at the time of colonoscopy; (2) patients receiving antibiotic treatment from three months prior to the study until its completion; (3) patients who had travelled to developing or underdeveloped countries from three months before the start of the study until its completion; (4) patients on low-calorie diets, vegan diets, gluten-free diets and other ‘special’ diets; (5) patients consuming probiotics or herbal remedies from three months before the start of the study until its completion; (6) bacterial or parasite intestinal infection (including Blastocystis hominis) in the previous three months; (7) previous history of coeliac disease, inflammatory bowel disease or other types of enteropathy; (8) previous gastrointestinal surgery (excluding appendectomy or inguinal herniorrhaphy); (9) alcoholism or drug addiction; and (10) inability to understand the instructions for participating in this study.

Healthy volunteers aged 18–75 years without digestive symptoms and none of the exclusion criteria above described were included after signing the informed consent and formed the healthy control group (HC).

#### Faecal sample collection

In all included patients, faecal samples were collected both three days before split-dose PEG colonic cleansing and at 30 days after colonoscopy (always before starting specific treatment for the diarrhoeal illness). Faecal samples were collected by the patients at home in appropriate sterile plastic containers and immediately frozen at − 20 °C. To avoid sample thawing, portable thermal systems were used to transport the faecal samples from home to the laboratory. The samples were then stored at − 80 °C until processing.

Healthy volunteers also performed PEG colonic cleansing and collected faecal samples following the same protocol: 3 days before PEG and 30 days after PEG. The same procedure for sampling, freezing, and storing faecal samples was used.

#### Diagnostic work-up of chronic watery diarrhoea

A complete colonoscopy was performed under conscious intravenous sedation on all included patients. Multiple biopsy specimen samples were obtained when the macroscopic appearance of the colonic mucosa was normal or mildly abnormal (mild erythema or oedema). Routinely, four samples from the ascending colon, and two each from the transverse, descending, and sigmoid zones, were taken. MC diagnosis was based on both clinical and histological criteria as previously defined [1, 2]. Histological MC diagnosis was reviewed in all cases by experienced pathologists at the participating centres.

When histological examination of colonic samples was normal and diarrhoea persisted, a ^75^SeHCAT (Se-homotaurocholate) abdominal retention test was performed to assess BAD [14]. BAD was defined as a seventh day retention value < 10%. A value < 5% was considered to be severe BAD. Capsule endoscopy or intestinal MRI was performed on patients with normal ileocolonoscopy and increased levels of faecal calprotectin to completely rule out either Crohn’s disease or other small bowel enteropathies. FD and diarrhoea-predominant IBS were diagnosed when the results of all specific tests performed were normal and the patient fulfilled the Roma III criteria for each functional disease.

#### Clinical remission definition

Patients were visited at 30 days after colonoscopy. Clinical remission was defined as the absence of watery stools (Bristol scale ≤ 5) in the last week before visit. Patients were followed up by a phone call at month four to rule out diarrhoea relapse.

### Faecal microbiome analysis

#### Genomic DNA extraction

DNA was extracted following the International Human Microbiome Standards (IHMS; http://www.microbiome-standards.org) [15]. A frozen aliquot (250 mg) of each sample was suspended in 250 mL of guanidine thiocyanate, 40 mL of 10% N-lauroyl sarcosine and 500 mL of 5% N-lauroyl sarcosine. DNA was extracted by the mechanical disruption of the microbial cells with beads and nucleic acids were recovered from clear lysates by alcohol precipitation. An equivalent of 1 mg of each sample was used for DNA quantification using a NanoDrop ND-1000 Spectrophotometer (Nucliber). DNA integrity was examined by micro-capillary electrophoresis using an Agilent 2100 Bioanalyzer.

#### High-throughput DNA sequencing

For profiling microbiome composition, the hyper-variable region (V4) of the bacterial and archaeal 16S rRNA gene was amplified by PCR. For amplification, the universal primers V4F_517_17: 5′GCCAGCAGCCGCGGTAA-3′ (Forward primer) and V4R_805_19: 5′-GACTACCAGGGTATCTAAT-3′ (Reverse primer) were used. The use of these primers guarantees the amplification of practically 100% of the domains of bacteria and archaea. The sequencing process, following standard Illumina platform protocols (Illumina website), was performed as previously described [16].

#### Sequence data analysis

The raw sequences were loaded into the QIIME 1.9.1 pipeline [16]. To study diversity information, Operational Taxonomic Units (OTUs) tables were performed. To estimate the microbial richness and evenness of the sample, in terms of what are known as alpha-diversity estimates, we calculated the Chao1 and Shannon diversity indexes [17, 18]. To calculate between-samples diversity or beta-diversity, weighted and unweighted UniFrac metrics were applied to build phylogenetic distance matrices, which were then used to construct hierarchical cluster trees using the Unweighted Pair Group Method with Arithmetic mean and Principal Coordinate Analysis (PcoA) representations.

#### Microbial dysbiosis index

The microbial dysbiosis index (MD-index) was calculated as previously described [19]. The MD-index is defined as the log of [total abundance in organisms increased in either MC, FD or BAD] over [total abundance of organisms decreased in either MC, FD or BAD]. Increased or decreased organisms were defined as those with a *p* < 0.05 in comparison with the HC group.

### Ethical issues

The study protocol was submitted and approved by the local Ethical and Research Committees of both the Hospital Universitari Mutua Terrassa (18 June 2014, Acta 06/14, Terrassa, Barcelona, Spain) and the University Hospital Vall d’Hebron (Barcelona, Spain). The study was conducted in accordance with the Declaration of Helsinki. All patients and healthy volunteers received information concerning their participation in the study and gave written informed consent.

### Statistical analyses

This was an exploratory observational study and no calculation of the sample size was intended. There were no previous data about the remission rate achieved after PEG colonic cleansing, and in this sense present study should be considered as a pilot study. We select the inclusion criteria to maximize the pre-test probability of MC and we stopped patient recruitment when 20 MC patients were included.

Statistical analyses were carried out in QIIME and in R [16]. To work with normalised data, we analysed an equal number of sequences from all groups. The Shapiro–Wilk test was used to check the normality of data distribution. Parametric normally distributed data were compared by the Student’s *t* test for paired or unpaired data. Otherwise, the Wilcoxon signed rank test was used for paired data and the Mann–Whitney U test for unpaired data. The Kruskal–Wallis one-way test of variance was used to compare the median alpha-diversity and MD-Index between groups, as well as the number of sequences of the groups at various taxonomic levels. The Friedman test was used for one-way repeated measures analysis of variance. We used a permutational multivariate analysis of variance (PERMANOVA), a non-parametric multivariate analysis of variance, to test for differences in microbial community composition adjusted for age and sex. When possible, the analysis provided false discovery rate (FDR)-corrected *p* values. FDR, q < 0.05 was considered significant. Spearman correlation was used to evaluate significant associations between alpha-diversity, MD-index and the daily stool number. MedCalc statistical software, version 18.2.1 (MedCalc Software bvba, Ostend, Belgium) was used to construct the figures.

## Results

### Clinical remission after colonoscopy

Fifty-five consecutive patients with chronic non-bloody watery diarrhoea were included (age, 62.0 ± 1.5 years; sex, 87.3% female). Clinical remission after colonoscopy was observed in 10 out of 19 (52.6%) patients diagnosed with MC (7 CC and 12 LC), and in 12 out of 36 (32%) patients with non-MC diarrhoea (*p* = 0.16). The final diagnostic work-up of the 24 non-MC patients with persisting diarrhoea after colonoscopy revealed that 17 (70.8%) of these patients had BAD (47% severe BAD), and seven had FD.

At the fourth month phone visit, eight out of 10 MC patients maintained clinical remission without requiring specific treatment. All patients with non-MC diarrhoea achieving remission maintained clinical remission at month four and were considered as FD for the study of faecal microbiota.

Three MC patients (1 CC, 2 LC), one patient with BAD and eight with FD either did not collect faecal samples or did not deliver them in appropriate conditions and were excluded from the study of faecal microbiota. Table 1 describes the baseline clinical characteristics of the patients included in the study of faecal microbiota as compared to HC.

**Table 1:** Baseline clinical characteristics of the diarrhoeal groups and healthy controls included in the study of faecal microbiota

Variable	MC (n = 16)	BAD (n = 16)	FD (n = 11)	HC (n = 12)	*p* value
Age (years)*	61.8 ± 2.9	57.5 ± 4.8	62.8 ± 3.7	42 ± 3.5	< 0.01
Sex (% female)	13 (81%)	12 (75%)	11 (100%)	9 (75%)	0.35
BMI*	24.7 ± 1.2	30.2 ± 1.7	25 ± 2.3	–	0.033
Total daily stool number**	5 (3–7.5)	4 (3.25–5)	3 (3–5)	–	0.35
Liquid daily stool number**	4.5 (2–5.5)	2 (2–3)	2 (2–3)	–	0.09
Diarrhoea duration (weeks)**	20 (8–156)	48 (42–156)	48 (32–96)	–	0.16
IBP usage (%)	5 (31%)	4 (25%)	4 (36%)	–	0.81
NSAID usage (%)	3 (18.7%)	2 (12.5%)	4 (36%)	–	0.31

*Mean (SEM); **Median (IQR)

The evolution of both daily total stool number and daily liquid stool number in each group after the colonoscopy are described in Table 2.

**Table 2:** Daily stool number before and 1-month after diagnostic colonoscopy in the study groups

Variable	Daily total stool number			Daily watery stool number		
	Baseline	Post-PEG	*p* value	Baseline	Post-PEG	*p* value
MC (n = 16)	5 (3–7.5)*	2 (1–5)	0.0001	4.5 (2–5.5)	0 (0–2.5)	0.0006
Non-MC (n = 27)	4 (3–5)	3 (2–4.25)	0.01	2 (2–3)	1 (0–3)	0.006
- BAD (n = 16)	4 (3.25–5)	4 (3–5)	0.38	2 (2–3)	2 (1–3.75)	0.30
- FD (n = 11)	3 (3–5)	2 (1–3)	0.015	2 (2–3)	0 (0–0)	0.0078

*Median (IQR)

### Baseline microbial diversity

**Fig. 1:**
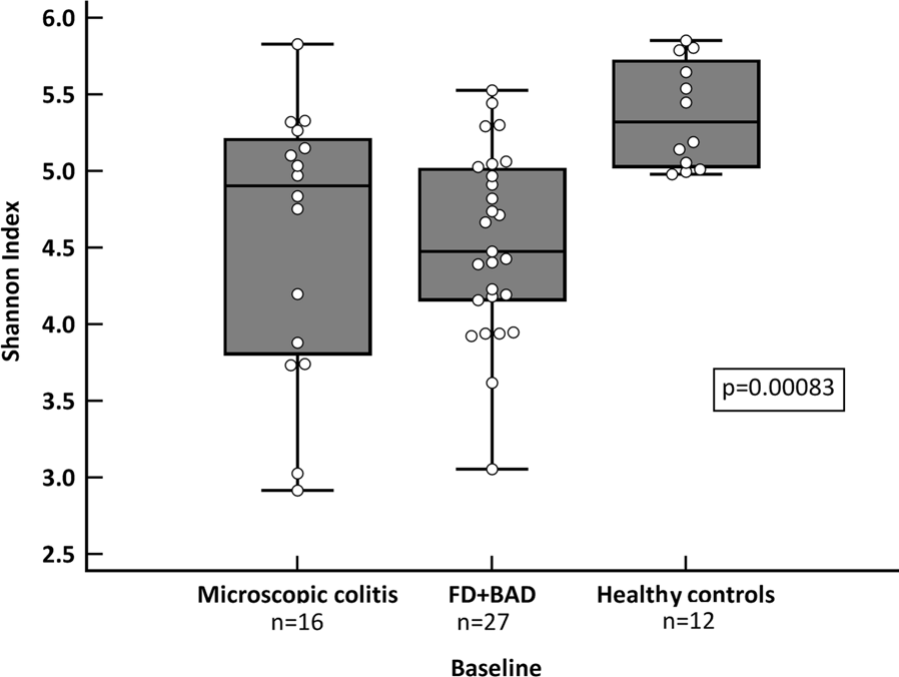
Boxplot describing the comparison of baseline alpha diversity (Shannon index) between patients with microscopic colitis (MC), functional diarrhoea *plus* bile acid diarrhoea (FD + BAD) and healthy controls

There was no significant difference in alpha-diversity (both Shannon and Chao1 indexes) between patients with MC, BAD and FD (Shannon, MC: median 4.9, IQR 3.8–5.2; BAD: median 4.7, IQR 3.9–5.0; FD: median 4.4, IQR 4.2–4.9; *p* = 0.84; Chao1, MC: median 236.3, IQR 227.5–255, BAD: median 214.5, IQR 170.5–249; FD: median 222.6, IQR 167–243.6; *p* = 0.33). The Shannon Index score was significantly lower in all diarrhoea groups as compared to healthy controls (Fig. 1; *p* = 0.0025). In addition, there was a non-significant trend to a lower Chao1 Index score in the diarrhoea groups as compared to healthy controls (Additional file 1: Fig. S1; *p* = 0.07).

We did not find differences in beta diversity level between BAD and FD groups at weighted or at unweighted UniFrac distances (*p* values 0.08 and 0.14, respectively), and there were no differences between MC and BAD + FD for weighted and unweighted UniFrac distances (*p* values 0.87 and 0.47, respectively) using a PERMANOVA test adjusted for sex and age (Additional file 1: Fig. S2).

There were no differences between CC and LC for both alpha diversity and beta diversity (data not shown).

There was no significant correlation between alpha-diversity and baseline daily stool number (Shannon index: Total stools, rho = − 0.008, *p* = 0.95; Watery stools, rho = 0.04; *p* = 0.81; Chao1 index: Total stools, rho = − 0.08, *p* = 0.65; Watery stools, rho = − 0.07; *p* = 0.69).

### Baseline bacterial composition

When applying FDR corrections, no bacterial species showed significantly different relative abundances between the study groups (BAD *versus* FD, or MC *versus* BAD + FD). However, there were differences between diarrhoea patients and healthy controls, as shown in Additional file 1: Table S1. Clostridiales (unclassified) were significantly decreased in MC as compared to healthy subjects (q = 0.042). No other bacteria were significantly different as compared to healthy controls after FDR correction, although there was a trend to decreased levels in many Firmicutes and some Actinobacteria and Bacteroidetes in patients with MC.

### Baseline microbial dysbiosis index

**Fig. 2:**
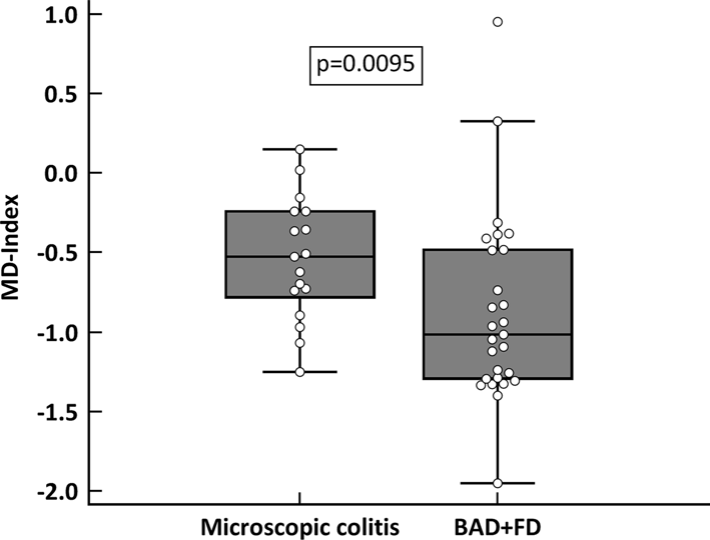
Boxplot describing the comparison of baseline microbial dysbiosis Index (MD-index) between patients with microscopic colitis (MC), and functional diarrhoea *plus* bile acid diarrhoea (FD + BAD)

Increases and decreases in bacteria (*p* < 0.05 in the non-adjusted analysis for multiple comparisons) as compared to healthy controls in each group are described in Additional file 1: Table S2. There were no differences between BAD and FD groups, which were therefore pooled together. The MD-index was significantly higher in MC than in BAD + FD (Fig. 2; *p* = 0.0095). There was no difference between CC and LC in terms of the MD-Index (data not shown).

There was no significant correlation between MD-Index and baseline daily stool number (Total stools: rho = 0.17, *p* = 0.44; Watery stools: rho = 0.24; *p* = 0.25).

### Effect of polyethylene glycol colonic lavage on faecal microbiome

**Fig. 3:**
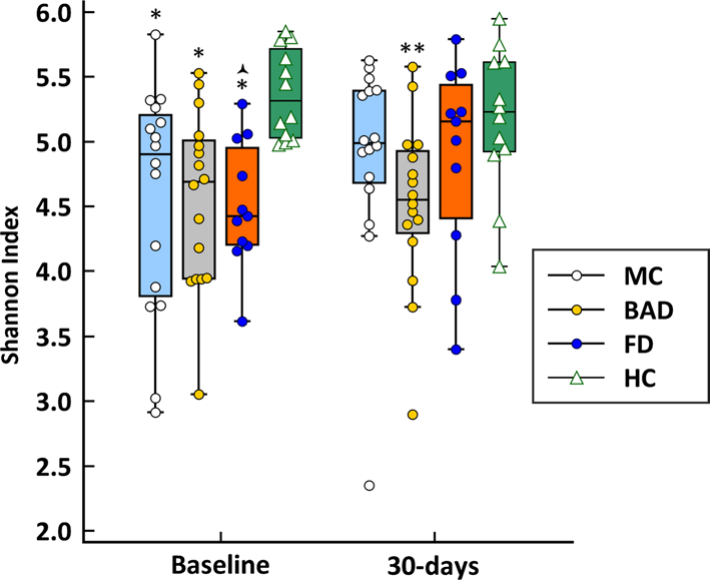
Boxplots describing the evolution of Shannon index 30-days after colonoscopy in both diarrhoeal groups and healthy controls (Repeated measures ANOVA: *p* = 0.015; *p = 0.0025 vs. HC; **p = 0.049 vs. HC; *p* = 0.03 vs. 30-days) (*MC* microscopic colitis, *FD* functional diarrhoea, *BAD* bile acid diarrhoea, *HC* healthy controls)

The evolution of alpha-diversity in diarrhoeal groups and HC after PEG is described in Fig. 3. In the HC group there were no significant differences in alpha-diversity at 30 days after PEG as compared to baseline (both Shannon and Chao1 indexes). Shannon index scores increased in MC and FD patients at 30 days after colonoscopy, but the differences only were significant for the latter (*p* = 0.03). Only in the BAD group did Shannon index scores remained significantly lower than in HC at 30 days of follow-up (*p* = 0.049). There were no significant differences in the Chao1 index (data not shown). In addition, there were no differences in the percentage change in Shannon index scores between MC patients with either clinical remission or persisting diarrhoea (data not shown).

**Fig. 4:**
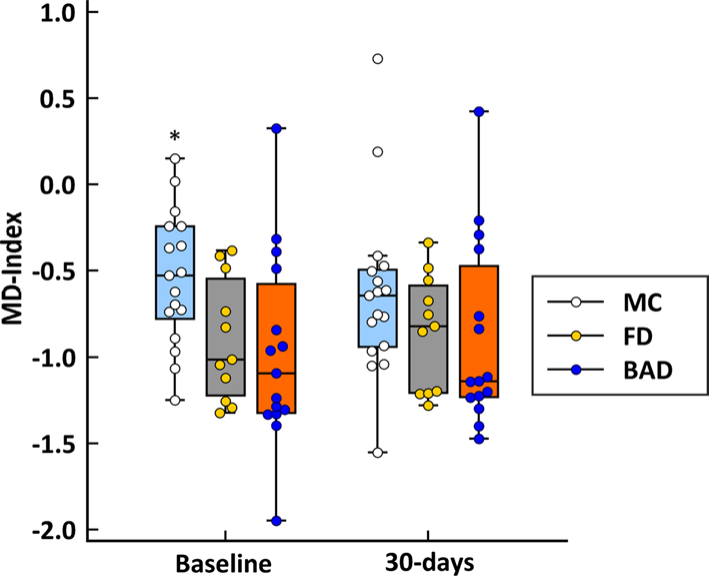
Boxplots describing the evolution of microbial dysbiosis index 30-days after colonoscopy in diarrhoeal groups (*MC* microscopic colitis, *FD* functional diarrhoea, *BAD* bile acid diarrhoea). *p = 0.014 vs. FD and BAD

The evolution of MD-index scores after PEG is described in Fig. 4. There were no significant differences between groups when comparing baseline *versus* the 30-days follow-up. However, whereas the MD-index was higher in MC patients as compared to the other two groups (*p* = 0.014) at baseline, this statistical significance was lost at the 30-day measurement. In addition, there were no differences in the MD-index percentage change between MC patients with either clinical remission or persisting diarrhoea (data not shown).

There were no changes in operative taxonomic units (OTUs) for each group comparing baseline *versus* 30-day measurements at *p* value < 0.01. Differences at *p* value < 0.03 are described in Additional file 1: Table S3 (N.S. at q-value < 0.05).

## Discussion

This is the first study with a design that permits the assessment of gut microbiota in active MC before diagnostic colonoscopy, thus avoiding changes in the microbiome induced by colonic preparation. The results obtained show that the reported changes in alpha and beta-diversity in active MC are unspecific and are shared by other chronic watery diarrhoeal diseases such as FD (Rome III criteria) and BAD when compared to healthy controls. In fact, there is a well-known association between stool form (as measured by the Bristol stool scale) and species richness, which reaches its minimum in diarrhoea-afflicted subjects [20]. However, MD-index was higher in MC as compared to both FD and BAD. Taxa from the Firmicutes phylum (Clostridiales) were more often decreased in the three diarrhoeal groups, but an unidentified member of the Clostridiales order significantly decreased at the FDR level only for MC. These findings may be related to more severe diarrhoea in the MC group, which presented with more daily liquid stools.

Noteworthy, a considerable number of MC achieved clinical remission after the colonoscopy, suggesting that PEG-induced gut microbiome changes may play a role. In contrast, most non-MC patients with persisting diarrhoea after PEG were diagnosed from BAD, indicating a lower clinical remission rate in these patients after PEG. Thirty days after colonoscopy, there was an increase in Firmicutes (Clostridiales) in both MC and FD, but not in BAD. This partial gut microbiota recovery 30 days after PEG lavage had previously been described in healthy controls [21]. Consequently, MD-index improved at 30-days in MC patients but not in BAD patients.

PEG-based colonic preparations remove intestinal mucus and endoluminal bacteria quite effectively, disrupting the balance of the microbiota. Besides, intestinal preparations carry oxygen into the lumen of the colon, which negatively affects anaerobic bacterial populations. Also, the availability of nutrients (especially dietary fibre and other fermentable carbohydrates) specific for bacterial metabolism is reduced, and intestinal transit time is accelerated. All these factors can rapidly change the composition of the gut microbiota and its homeostasis [13]. Most studies have addressed the gut microbiota modifications associated with bowel cleansing immediately after colonoscopy, or the next day. Very few data have been reported concerning potential side-effects related to changes in microbiome composition after a longer period. Some of these studies have described the development of minor abdominal pain and distension after the endoscopic procedure persisting at the 30 days follow-up [22–24], which could be prevented by using supplementation with probiotics [25, 26]. In addition, it has been stated that PEG-lavage could induce mild clinical relapses in ulcerative colitis [27]. Thus, it seems paradoxical than PEG-colonic lavage may have positive implications too. As mentioned previously, luminal factors play a not well-known role in the pathogenesis of MC. In this sense, it could be hypothesized that PEG-lavage is able to change this luminal milieu and even the mucosa-adhering bacteria, thus promoting clinical remission. Regrettably, we could not demonstrate a related PEG-induced change in faecal microbiome. These observations warrant further studies on the effect of PEG lavage on mucosal-adhering bacteria and the immune mechanisms of mucosal inflammation in MC.

The differences observed in BAD patients are striking. In these subjects, the pathogenesis of diarrhoea is prompted by the irritating effect of primary bile acids on colonic mucosa, promoting secretion and the motility of the colon [28]. It has been suggested the existence of a gut microbiota–driven mechanism, specifically Clostridia-rich microbiota-driven excessive bile acid synthesis and excretion in the pathogenesis of BAD [29]. However, the present results show that there is a decrease in Clostridiales abundance like MC and FD in BAD patients. In fact, we did not find any significant difference in gut microbioma between BAD and FD at baseline. However, in another previous study [30], BAD patients experienced a decrease in alpha-diversity, though a greater abundance of certain anaerobic taxa, including specific members of Lachnospiraceae, *Bifidobacteria*, *Prevotella*, Verrucomicrobia and *Bacteroides* was found as compared to IBS-D. The difference between the present study and those previous studies could be in the severity of BAD. In one study, BAD was investigated by means of the total bile acid concentration in a single stool sample and it is not clear how correlates this test with the 7-day SeHCAT test [29], and in a second study there were clear differences in the severity of BAD with a cut-off of normality < 15% [30], instead of the 10% used in the present study, being moderate/severe in 85% and 100% of patients, respectively. In any case, it is unclear whether the persistence of the same degree of diarrhoea 30 days after PEG-lavage associated with persisting changes in microbial diversity and dysbiosis observed in the present study are the effects of the higher concentrations of bile acids entering the colon or if the microbiota changes are also a causative factor in the development of BAD.

A major strength of our study is that for the first time, as compared to previous studies, faecal samples were obtained before the colonoscopy and thus were not modified by the bowel cleansing procedure. In addition, we used two diarrhoeal control groups to evaluate whether the microbiota changes were associated with diarrhoea per se. However, we acknowledge several limitations. Our sample size limited our power to identify modest compositional associations. More information would have been obtained with larger numbers. However, our findings help to identify areas for further confirmatory studies. The use of faecal samples is another possible limitation, since it can be assumed that the mucosal microbiome is important in MC. Also, the use of 16S rRNA sequencing may have negatively affected our ability to identify rare taxa, because the technique precludes the ability to detect low abundant taxa. Thus, the absence of MC-specific taxa must be interpreted with caution. Age, sex, and BMI have been associated with gut microbiota composition and identified as important potential confounders. To rule out the possibility that the results were confounded by age and sex, they were examined by a multivariate analysis, though our sample size did not allow us to adjust for other potential confounders. Finally, we did not conduct dietary questionnaires during the study, and we cannot rule out an effect of different dietary intake on intestinal microbiota, but presumably it should not be very different in the diarrhoea groups.

In conclusion, dysbiosis is a feature in active MC, but faecal microbial diversity was similarly reduced in MC, BAD and FD, suggesting that faecal microbial changes are not due to MC itself but associated with stool form. A considerable number of MC patients achieved clinical remission after colonoscopy, but we could not demonstrate related PEG-induced changes in faecal microbiome. In contrast, diarrhoea persisted in most patients with BAD, which was associated with ongoing dysbiosis. Further studies are required to study mucosal adhering bacteria and their interplay with the immune function in MC patients.

## Supplementary Information

**Additional file 1.** Supplemental Data.

## Data Availability

DNA sequences were uploaded in the NCBI repository with the following accession number: https://www.ncbi.nlm.nih.gov/bioproject/PRJNA840848.

## Abbreviations

MC: Microscopic colitis
PEG: Polyethylene glycol
BAD: Bile acid diarrhoea
FD: Functional diarrhoea
LC: Lymphocytic colitis
CC: Collagenous colitis
HC: Healthy controls
MD-index: Microbial dysbiosis index
