# Supplementary material for: Colonic bacterial diversity and dysbiosis in active microscopic colitis as compared to chronic diarrhoea and healthy controls: effect of polyethylene glycol after bowel lavage for colonoscopy
Source: BMC Gastroenterol. 2022 Jun 28;22:320. doi: 10.1186/s12876-022-02392-w (PMC9238263; doi:10.1186/s12876-022-02392-w)

**SUPPLEMENTAL DATA**

**Table 1 suppl.** Changes in relative abundances of operative taxonomic units (OTUs) in groups with diarrhea as compared to healthy controls that showed significant differences at an uncorrected p value <0.01: A, Actinobacteria; B, Bacteroidetes; F, Firmicutes; P, Proteobacteria (*q<0.05). Arrows indicate if there was increase or decrease of the OTUs as compared to healthy controls.

| Taxonomy | P value |
| --- | --- |
| Microscopic colitis *vs.* healthy |  |
| A/Actinobacteridae/Bifidobacteriales/Bifidobacteriaceae/*Bifidobacterium adolescentis* | ↓0.003 |
| A/Coriobacteridae/Coriobacterineae/Coriobacteriaceae/unclassified/ | ↓0.0068 |
| B/Bacteroidia/Bacteroidales/Bacteroidaceae/*Bacteroides*/ | ↓0.009 |
| B/Bacteroidia/Bacteroidales/Bacteroidaceae/*Bacteroides*/ | ↓0.005 |
| F/Clostridia/Clostridiales/unclassified/ | ↓0.00015* |
| F/Clostridia/Clostridiales/Lachnospiraceae/unclassified/ | ↓0.005 |
| F/Clostridia/Clostridiales/Lachnospiraceae/unclassified/ | ↓0.009 |
| F/Clostridia/Clostridiales/Lachnospiraceae/unclassified/ | ↓0.009 |
| F/Clostridia/Clostridiales/Lachnospiraceae/*Coprococcus*/ | ↓0.009 |
| F/Clostridia/Clostridiales/Lachnospiraceae/*Coprococcus*/ | ↓0.002 |
| F/Clostridia/Clostridiales/Ruminococcaceae/unclassified/ | ↓0.0023 |
| F/Clostridia/Clostridiales/Ruminococcaceae/*Ruminococus*/ | ↓0.0009 |
| F/Erysipelotrichia/Erysipelotrichales/Erysipelotrichaceae/*Catenibacterium*/ | ↓0.0066 |
| F/Erysipelotrichia/Erysipelotrichales/Erysipelotrichaceae/*Catenibacterium*/ | ↓0.007 |
| Other (non-identified) | ↑0.000009* |

| **Bile acid diarrhea *vs.* healthy** |  |
| --- | --- |
| A/Actinobacteridae/Bifidobacteriales/Bifidobacteriaceae/*Bifidobacterium adolescentis* | ↓0.0058 |
| A/Coriobacteridae/Coriobacterineae/Coriobacteriaceae/unclassified | ↓0.0092 |
| A/Coriobacteridae/Coriobacterineae/Coriobacteriaceae/unclassified | ↓0.0093 |
| B/Bacteroidia/Bacteroidales/Bacteroidaceae/*Bacteroides*/unclassified | ↓0.0091 |
| B/Bacteroidia/Bacteroidales/Rikenellaceae/unclassified/ | ↓0.0087 |
| F/Clostridia/Clostridiales/unclassified/ | ↓0.0007 |
| F/Clostridia/Clostridiales/Ruminococcaceae/*Oscillospira*/ | ↓0.0021 |
| F/Clostridia/Clostridiales/unclassified/ | ↓0.0031 |
| F/Clostridia/Clostridiales/Ruminococcaceae/unclassified/ | ↓0.0044 |
| F/Clostridia/Clostridiales/Ruminococcaceae/unclassified/ | ↓0.0048 |
| F/Clostridia/Clostridiales/Ruminococcaceae/unclassified/ | ↓0.0060 |
| F/Clostridia/Clostridiales/Clostridiaceae/unclassified/ | ↓0.0071 |
| F/Clostridia/Clostridiales/unclassified/ | ↓0.0072 |
| F/Clostridia/Clostridiales/Clostridiaceae/unclassified/ | ↓0.0079 |
| F/Clostridia/Clostridiales/Ruminococcaceae/*Faecalibacterium prausnitzii* | ↓0.0093 |
| P/Gammaproteobacteria/Enterobacteriales/Enterobacteriaceae/*Escherichia coli* | ↑0.0058 |
| Other (non-identified) | ↑0.000009* |
| **Functional diarrhea *vs.* healthy** |  |
| B/Bacteroidia/Bacteroidales/Bacteroidaceae/*Bacteroides*/unclassified/ | ↓0.0013 |
| B/Bacteroidia/Bacteroidales/Bacteroidaceae/*Bacteroides*/unclassified/ | ↓0.0015 |
| F/Clostridia/Clostridiales/Ruminococcaceae/unclassified/ | ↓0.0009 |
| F/Clostridia/Clostridiales/Ruminococcaceae/unclassified/ | ↓0.0041 |
| F/Clostridia/Clostridiales/Lachnospiraceae/*Coprococcus*/ | ↓0.0046 |
| F/Clostridia/Clostridiales/Lachnospiraceae/*Coprococcus*/ | ↓0.0054 |
| F/Clostridia/Clostridiales/Lachnospiraceae/unclassified/ | ↓0.0061 |
| F/Clostridia/Clostridiales/Lachnospiraceae/unclassified/ | ↓0.0067 |
| F/Clostridia/Clostridiales/unclassified/ | ↓0.0072 |
| F/Clostridia/Clostridiales/Lachnospiraceae/unclassified/ | ↓0.0088 |
| F/Clostridia/Clostridiales/Lachnospiraceae/unclassified/ | ↓0.0097 |
| Other (non-identified) | ↑0.00005* |

**Table 2 suppl.** Increased and decreased bacteria as compared to healthy controls in each group, used to calculate the microbial dysbiosis index (p<0.05 in the non-adjusted analysis for multiple comparisons).

| **Groups** | **Increased bacteria** | **Decreased bacteria** |
| --- | --- | --- |
| Microscopic colitis | *Clostridium perfringens*  *Clostridium saccharogumia*  *Dialister sp*  *Veillonella parvula*  *Citrobacter sp*  Others (non-identified) | *Bifidobacterium adolescentis*  Coriobacteriaceae  Bacteroidaceae  Porphyromonadaceae  Rikenellaceae  Clostridiaceae  Lachnospiraceae  Ruminococcaceae  Erysipelotrichaceae  Acidaminococcaceae  Succinivibrionaceae |
| Bile acid diarrhoea | *Escherichia coli*  Others (non-identified) | Clostridiaceae  Ruminococcaceae (*Faecalibacterium prausnitzii, Oscillospira sp,* others)  *Bifidobacterium adolescentis*  Rikenellaceae  Bacteroidaceae (*B plebeius,* others)  Coriobacteriaceae  Streptophyta  *Prevotella sp*  Peptostreptococcaceae  Lachnospiraceae (*Coprococcus sp, Blautia sp*, others)  Christensenellaceae  Erysipelotrichaceae  Succinivibrionaceae  Alphaproteobacteria |
| Functional diarrhoea | Others (non-identified) | Bifidobacteriaceae (*B adolescentis*)  Bacteroidaceae  Rikenellaceae  Porphyromonadaceae  Ruminococcaceae  Lachnospiraceae  Peptostreptococcaceae  Clostridiaceae  Erysipelotrichaceae  Veillonellaceae  Lachnospiraceae (*Blautia sp, Dorea sp*)  Acidaminococcaceae  Sutterellaceae  Coriobacteriaceae  Methanobacteriaceae |

**Table 3 suppl.** Changes in relative abundance of operative taxonomic units (OTUs) at 1-month as compared to baseline in groups with diarrhea (only p values <0.03 are listed). F, Firmicutes; P, Proteobacteria (no bacteria was significant at q value <0.05). Arrows indicate if there was increase or decrease of the OTUs as compared to healthy controls.

| **Taxonomy** | **Basal** | **1-month** | **p-Value** |
| --- | --- | --- | --- |
| ***MC:*** |  |  |  |
| F/Clostridia/Clostridiales/Lachnospiraceae/*Lachnospira* | 0.0014±0.0064 | 0.006±0.015 | ↑0.014 |
| ***FD:*** |  |  |  |
| F/Clostridia/Clostridiales/Lachnospiraceae/*Dorea* | 0.00078±0.001 | 0.005±0.006 | ↑0.013 |
| F/Clostridia/Clostridiales/Ruminococcaceae/unclassified | 0.0003±0.0005 | 0.0011±0.001 | ↑0.022 |
| F/Clostridia/Clostridiales/Ruminococcaceae/*Ruminococcus* | 0.00029±0.0004 | 0.00058±0.005 | ↑0.022 |
| F/Clostridia/Clostridiales/Ruminococcaceae/*Oscillospira*/ | 0.000098±0.0003 | 0.0002±0.0005 | ↑0.024 |
| ***BAD:*** |  |  |  |
| P/Deltaproteobacteria/Desulfovibrionales/Desulfovibrionaceae/ *Bilophila* | 0.0003±0.003 | 0.0001±0.0003 | ↓0.027 |

**Figure 1 suppl.** Boxplot describing the comparison of baseline alpha diversity (Chao1 Index) between patients with microscopic colitis (MC), functional diarrhoea *plus* bile acid diarrhoea (FD+BAD) and healthy controls.


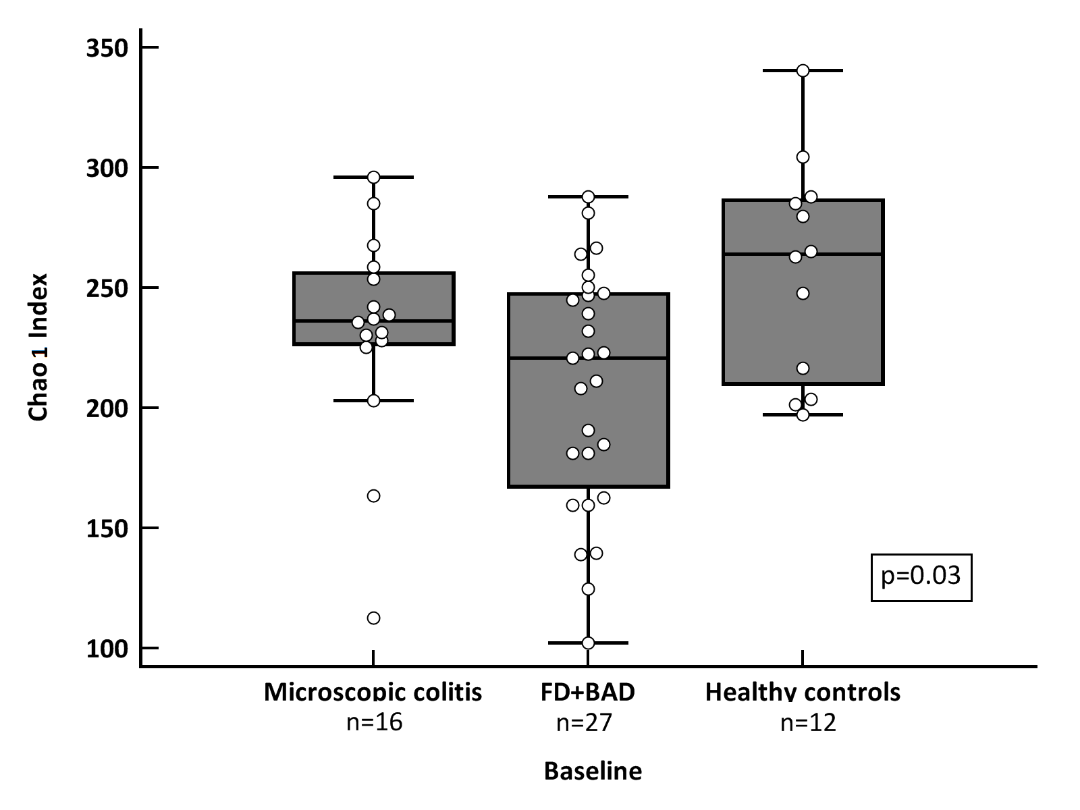


**Figure 2 suppl**. Comparison of baseline beta diversity between patients with microscopic colitis (MC) and bile acid diarrhoea *plus* functional diarrhoea (BAD+FD). A- weighted and B- unweighted UniFrac distances.

(P-values 0.87 and 0.47 respectively, using a PERMANOVA test adjusted for sex and age).

**A-**


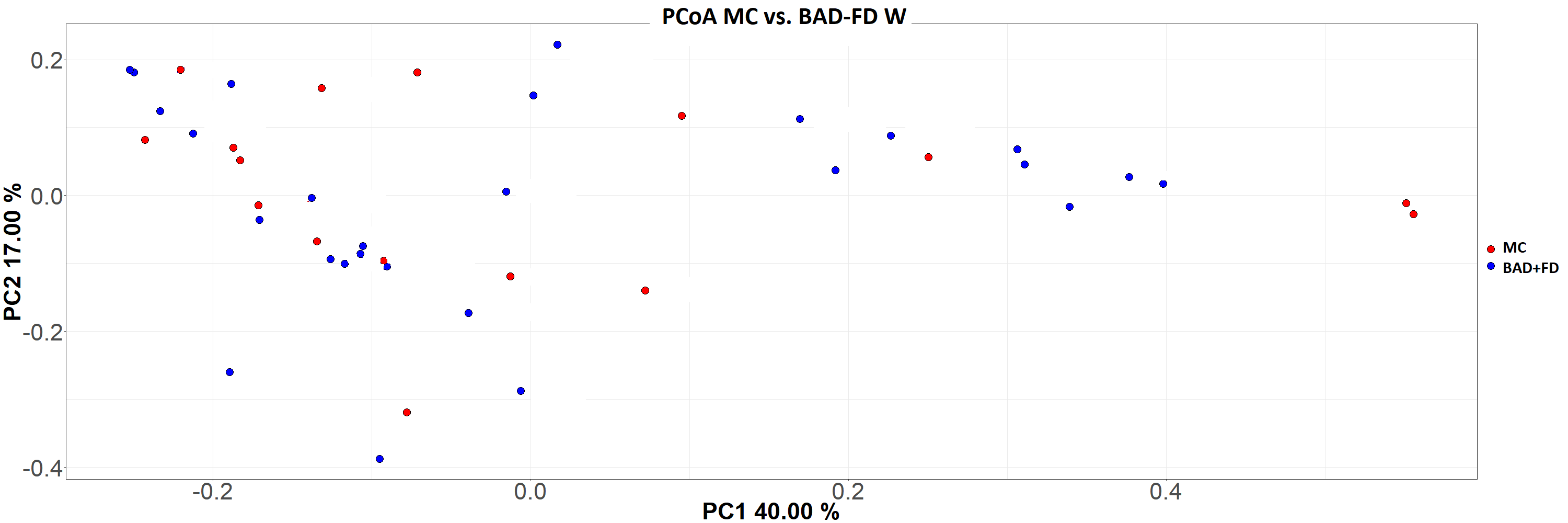


**B-**


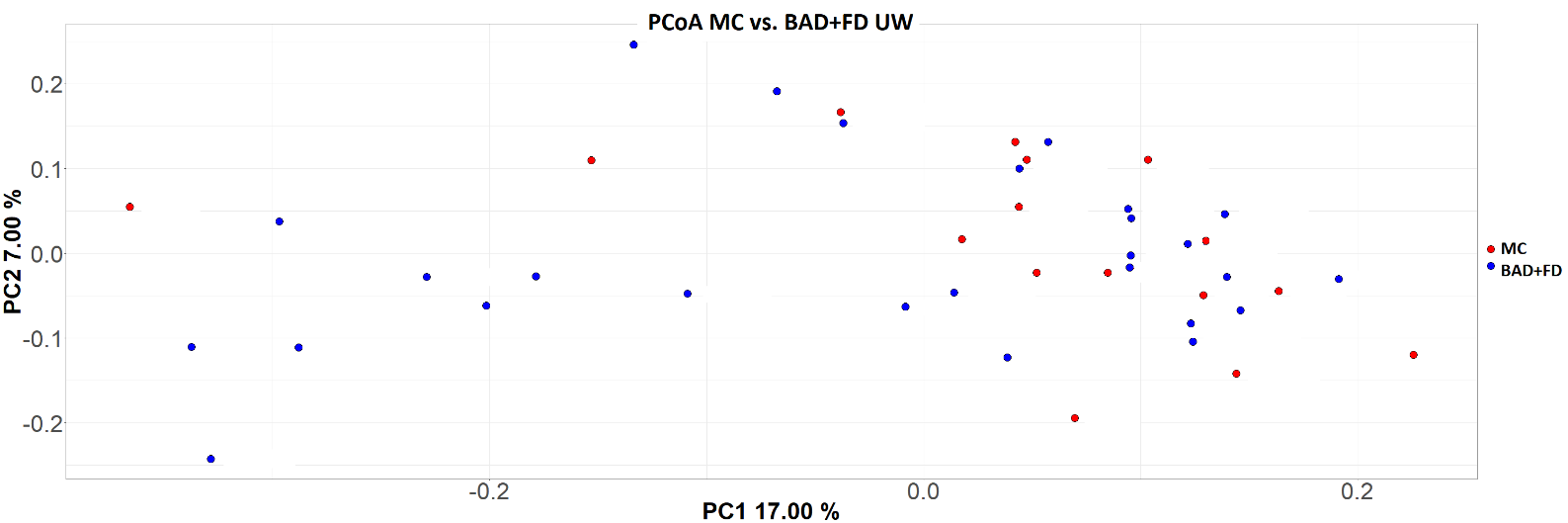

Supplement: Supplementary file 1 — Additional file 1. Supplemental Data. [file 12876_2022_2392_MOESM1_ESM.docx]
